# Carbothermal Synthesis of Nitrogen-Doped Graphene Composites for Energy Conversion and Storage Devices

**DOI:** 10.3389/fchem.2018.00501

**Published:** 2018-10-22

**Authors:** Hongwei Mi, Xiaodan Yang, Jun Hu, Qianling Zhang, Jianhong Liu

**Affiliations:** College of Chemistry and Environmental Engineering, Shenzhen University, Shenzhen, China

**Keywords:** liquid-polyacrylonitrile (LPAN), carbothermal reduction, template activated method, supercapacitors, lithium-ion battery

## Abstract

Metal oxides and carbonaceous composites are both promising materials for electrochemical energy conversion and storage devices, such as secondary rechargeable batteries, fuel cells and electrochemical capacitors. In this study, Fe_3_O_4_ nanoparticles wrapped in nitrogen-doped (N-doped) graphene nanosheets (Fe_3_O_4_@G) were fabricated by a facile one-step carbothermal reduction method derived from Fe_2_O_3_ and liquid-polyacrylonitrile (LPAN). The unique two-dimensional structure of N-doped graphene nanosheets, can not only accommodate the volume changes during lithium intercalation/extraction processes and suppress the particles aggregation but also act as an electronically conductive matrix to improve the electrochemical performance of Fe_3_O_4_ anode, especially the rate capability. What's more, by etching Fe_3_O_4_@G to remove the iron-based oxide template, porous N-doped graphene composites (NGCs) were prepared and presented abundant pore structure with high specific surface area, delivering a specific capacitance of 172 F·g^−1^ at 0.5 A·g^−1^. In this way, Fe_2_O_3_ was both template and activator to adjust the pore size of graphene. And the effect of specific surface area and pore size tuned by the Fe_2_O_3_ activator were also revealed.

## Introduction

Due to increasing energy and environmental demands, the utilization of energy storage devices have become a pressing essential need in both civil and military applications (Dunn et al., 2011; Etacheri et al., 2011; Chu and Majumdar, 2012; Li et al., in press). As materials play a leading role in the research of energy storage devices, metal oxides are considered as promising materials for electrochemical energy storage and conversion devices, such as secondary rechargeable batteries(Chen et al., 2017; Cui et al., 2018; Yi et al., 2018; Zhao et al., 2018; Zheng et al., 2018), fuel cells and electrochemical capacitors (Jiang et al., 2012; Wang et al., 2012, 2016; Wu et al., 2012; Nithya and Arul, 2016). Among various metal oxides, Fe_3_O_4_ is extensively studied as an alternative electrode material for LIBs, with advantages of low cost, natural abundance, high electronic conductivity and high capacity (926 mAh·g^−1^; Huang et al., 2017; Liu et al., 2017; Wang et al., 2018; Yan et al., 2018). However, its practical application is hindered, because of huge volume change during cycle processes which resulted in severe capacity losses as well as electrode pulverization (Zhu et al., 2011a; Wu et al., 2013).

In order to maintain the electrode integrity, some strategies including coating with carbonaceous materials (He C. et al., 2013) and fabricating nanostructure (Behera, 2011; Lim et al., 2012; Zeng et al., 2014) have been widely reported. Nevertheless, to realize these improvements, many *in situ* synthetic methods, such as sol-gel polymerization (Jung et al., 2013), solvothermal or hydrothermal method (Yuan et al., 2011; Zhu et al., 2011a), electrospinning (Wang et al., 2008) and chemical vapor deposition (Zhu et al., 2013) have been utilized, but they are short for large-scale application. What' more, carbonaceous materials especially graphene, which received worldwide attention owing to its outstanding properties, shows superior performances in high-performance lithium-ion batteries due to their good conductivity and large surface areas (Behera, 2011; Yan et al., 2014). In this regard, it is an effective approach for coating graphene on Fe_3_O_4_ to improve conductivity and relieve the volume change during cycles at the same time.

Besides batteries, carbonaceous materials also draw great attention as the electrode of the electrochemical double layer capacitors (EDLC) (Zhang and Zhao, 2009). Generally, to obtain high-performance EDLC electrode materials, there are usually several factors to consider. First of all, the specific surface area can greatly determine the capacitance of carbon (Zhao et al., 2017). In this respect, the fabrication of hollow (Han et al., 2014; Xu et al., 2015) or porous structure is an effective way to obtain the carbonaceous materials with large specific surface area. For example, Zhao et al. (Zhao et al., 2017) reported a novel 3D hierarchical carbon-based nanocages prepared by *in-situ* Cu template method, which can relieve the inevitable π-π aggregation and restacking of graphene sheets. Apart from the specific surface area, pore distribution has a vital influence on the capacity. On the one hand, by increasing the proportion of micropore in the material, the specific capacity of the material increased significantly. KOH activation is a popular method to prepare microporous carbons to achieve higher capacitance (Zhu et al., 2011b; Zheng et al., 2015). On the other hand, when the micropore volume increases to a certain extent, the resistance of ions transport to the porous carbon channel increases, resulting in poor capacitance at high current density. Zheng et al. (2015) reported that mesopore could connect multiple micropores, and speed up the electrolyte ions transferring from the electrode surface to the materials, resulting in a greater extent microporous energy storage ability into full play. Porous carbon with abundant pore structure (micropore, mesopore, and macropore), perform excellent rate capacity. Nevertheless, porous carbon is usually prepared by a complex process with KOH activation and template method (Xing et al., 2009).

Herein, we developed a facile carbothermal reduction method to fabricate Fe_3_O_4_@N-doped graphene composites (Fe_3_O_4_@G) as anode for Li-ion battery. Derived from Fe_2_O_3_ and liquid polyacrylonitrile (LPAN), the Fe_3_O_4_@G can not only present enhanced conductivity but also accommodate the volume expansion of Fe_3_O_4_. In addition, after etching by hydrochloric acid (HCl) to remove the metal oxides template, porous N-doped graphene composites (NGCs) were obtained. Furthermore, the controllable preparation of porous graphene materials by template activated method was established. This approach takes some advantages. Firstly, Fe_2_O_3_ was not only raw material to transform to Fe_3_O_4_, but also a template and an activating agent to adjust the pore size of graphene in which we call it a template activated method. Secondly, as previously reported (Mi et al., 2014; Zhuo et al., 2014), the LPAN used in this paper is the reductant and graphene precursor, which shows two-dimensional structure of N-doped graphene nanosheets after a carbothermal process in flowing argon gas. Lastly, the carbothermal method is simple to operate, and is also an approach for large-scale production of composites for energy storage.

## Experimental

### Preparation of Fe_3_O_4_@G composites for Li-ion battery

Fe_2_O_3_ (5 g, Shanghai Lingfeng Chemical Reagent Co., Ltd., China) and LPAN (2 g) were mixed and stirred in ethanol for 4 h. Then the mixture was preoxidated in air at 220°C for 3 h and carbonized in an argon atmosphere at a series of temperature (500, 600, 700, 800, and 900°C) respectively for 4 h to prepared Fe_3_O_4_@G and N-doped graphene nanosheets (G). The as-prepared samples were named as Fe_3_O_4_@G-X, which X represented the carbonization temperature. As comparison, pure LPAN was also preoxidated in air at 220°C for 3 h and annealed in an argon atmosphere at 600°C. The product was named as G.

### Preparation of NGCs for supercapacitors

The as-prepared samples (Fe_3_O_4_@G-600, Fe_3_O_4_@G-700, Fe_3_O_4_@G-800 and Fe_3_O_4_@G-900) were treated with HCl solution (4 mol·L^−1^) for 48 h and repeatedly washed by de-ionized water. Finally, the products were dried in a vacuum oven at 90°C for 3 h. The final product was referred to as NGC600, NGC700, NGC800, or NGC900, corresponding to the carbonization temperature of 600, 700, 800, or 900°C. As comparison, pure LPAN was also cured in air at 220°C for 3 h and carbonized in an argon atmosphere at 700°C. The product was named as G700.

## Materials characterization

The morphology and structure of the samples were characterized by field emission scanning electron microscope (FESEM, JSM-7800F & TEAM Octane Plus, 15 kV) and a Tecnai G2 transmission electron microscope (TEM, FEI, USA). The crystalline structures were obtained by a D8 advance X-ray diffraction spectrometer (XRD, Bruker, Germany) using Cu Kα radiation. X-ray photoelectron spectroscopy (XPS) was carried out on the ESCAlab220iXL electron spectrometer from VG scientific using 300-W Al Kα radiation. Raman spectra were performed at the room temperature (inVia Reflex, Renishaw, UK). The specific surface area and pore size distributions of the samples were measured by BELSORP-MAX with N_2_ as absorbate at 77 K. All the samples were degassed at 150°C for 3 h before measurement. The specific surface area was obtained by the BET equation and the pore size distribution was estimated from the desorption branch of N_2_ isotherms by the BJH method. The thermogravimetric analysis (TG-DTA, Netzsch, Germany) was used to calculate the mass fraction of graphene.

## Electrochemical measurements for Li-ion battery

Mixtures, which consisted of 80 wt% active materials (Fe_2_O_3_, G, Fe_3_O_4_@Gs), 10 wt% carbon black (CB) and 10 wt% polyvinylidene fluoride (PVDF) dispersed in N-methyl pyrrolidinone (NMP), were pasted on copper foil. Then the coated Cu foil was dried at 100°C for 12 h and then cut into pieces with a diameter (φ) of 14 mm. The loading amount of active material was ~0.8 mg·cm^−2^. The electrochemical performances of the samples were tested using 2,032 coin-type cells, Celgard 2400 separator, 1 mol·L^−1^ LiPF_6_/EC:EMC:DMC (1:1:1 by volume) electrolyte, and Li-foil as the counter electrode in an Ar-filled glove box (MBRAUN, Germany) with oxygen and moisture contents of <0.1 ppm. Galvanostatic charge-discharge measurements were performed on a LAND-CT2001A battery test system (China) in a voltage range of 0.01–3.0 V (vs. Li^+^/Li) at various current densities. Cyclic voltammetry (CV) was evaluated at 0.1 mV·s^−1^ on a Solartron analytic 1470E cell test system in the range of 0.01–3.0 V. Electrochemical impedance spectroscopy (EIS) was conducted on a Solartron Impedance analyzer 1260A at an AC voltage of 10 mV amplitude from 100 kHz to 0.01 Hz.

## Electrochemical measurements for supercapacitors

Electrochemical performances were estimated by symmetric electrode-type coin cells. The fabrication of working electrodes was described as follow: the mixture of active materials, carbon black additive and PTFE emulsion (with a mass ratio of 85:10:5) were added in ethanol solvent. After a full stirring, the as-prepared slurry was coated on the nickel foam (φ14 mm) and dried at 80°C for 6 h in a vacuum oven. The loading amount of active material was 0.5–0.8 mg·cm^−2^. Finally, the working electrodes can be obtained by further pressing at a pressure of 8 MPa. Subsequently, two electrodes with similar loading mass were selected as electrodes, and separated by a cellulose membrane filled with 6 M KOH electrolyte. Cyclic voltammetry (CV), chronopotentiometry (CP), and electrochemical impedance spectroscopy (EIS) were performed by electrochemical workstation (CHI 670 C) at room temperature. Cycle performance was tested by the LAND CT2001A instrument. The EIS was conducted using a sinusoidal signal of 5 mV over the frequency range from 100 kHz to 0.01 Hz.

The corresponding specific capacitance is calculated by the following equation: (Xie et al., 2012; Wang et al., 2016).

$$\begin{array}{l} \text{Cs}=\frac{2I\Delta t}{mU} \end{array}$$

where *I* was the current, Δ*t* was the discharge time, *U* was the potential range, and *m* was the average mass of the samples on both electrodes.

## Results and discussion

**Figure 1:**
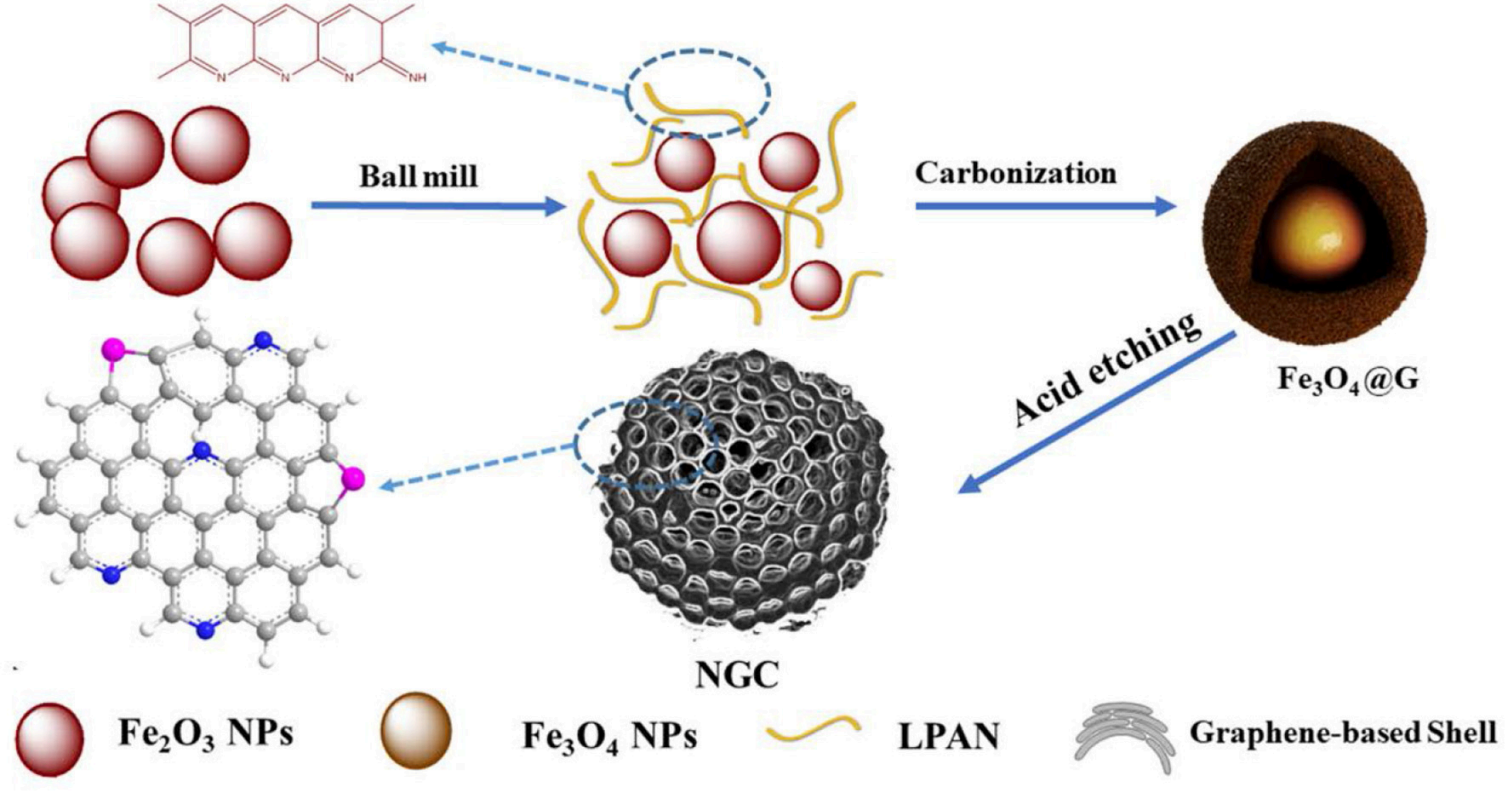
Schematic illustration of the formation route of Fe_3_O_4_@G and NGC.

As shown in Figure 1, Fe_3_O_4_@G was fabricated by a facile one-step carbothermal reduction method. Firstly, Fe_2_O_3_ powders were mixed with LPAN and stirred for 4 h in absolute ethyl alcohol solvent. Subsequently, the mixture was cured in air and carbonized for 4 h under argon flow. During carbonization, LPAN transformed to graphene, and Fe_2_O_3_ was reduced to Fe_3_O_4_. With the temperature increased, iron-based oxides (FeO_x_) further reacted with graphene, etching the graphene to increase the specific area as well as pore size. After treated with HCl solution, the FeO_x_ was removed, and NGC could be obtained.

## Carbothermal reduction method to prepare Fe_3_O_4_@G

The TG analysis and XRD were operated to reveal the reaction changes with various temperatures. TG curves shown in Figure S1A were measured from 30 to 1,000°C under the nitrogen flow. For the pure Fe_2_O_3_ sample, the TG curve was almost horizontal, indicating that thermal decomposition reactions didn't take place inside the Fe_2_O_3_ under 1,000°C except for the release of a little water. In the TG curve of pure LPAN, thermal decomposition reaction inside LPAN took place all the time because of the inherent nature of the organics. Before estimating by TG, Fe_2_O_3_/LPAN precursor was heated at 220°C in air for 3 h, to make the crosslinking reaction occurred inside the LPAN. There was a big mass break from 500 to 700°C in the mixtures. And the weight of samples didn't change until the temperature reaches 750°C, which indicated the reduction reactions inside the Fe_2_O_3_/LPAN precursors completed. As the Fe_3_O_4_@G was the intermediate of the reduction reactions, so the following carbothermal temperature will range from 500 to 700°C. LPAN is the oligomer of chained acrylonitrile and converts to graphene after carbonization (Mi et al., 2014; Zhuo et al., 2014). As shown in Figure S1B, the main diffraction peaks of the Fe_3_O_4_@G can be indexed to magnetite-based on their good agreement with JCPDS Card No. 88-0866, indicating the reduction reaction occurred from the Fe_2_O_3_/LPAN precursor (Zeng et al., 2014; Li et al., 2017). During carbonization, LPAN transforms into graphene, and Fe_2_O_3_ is mainly reduced to Fe_3_O_4_. With the temperature increased, Fe_3_O_4_ further reacted with carbon to form FeO and Fe, which we can also call activation (He X. et al., 2013).

According to the XRD results, when the temperature was below 600°C, Fe_2_O_3_ was mainly reduced to Fe_3_O_4_, which can be described as Equations (1,2). But if the temperature is 700°C, the other reduced phases including FeO (41.8°) (Equation 3) and Fe (44.8°) (Equation 4) were produced and the content of Fe_3_O_4_ will decrease accordingly. The equations of carbothermal reaction and activated reaction were given as followed (He X. et al., 2013). As the FeO phase and Fe phase show relatively inactive to Li^+^, the specific capacity of the prepared composites will decay quickly with the increased content of these new phases. Consequently, the carbothermal temperature should not exceed 700°C to avoid generating the inactive products. Herein, Fe_3_O_4_@G prepared at the temperature of 500, 600, and 700°C are used as anode for Li-ion battery.

(1) $$\begin{array}{r} {2\text{C}+\text{O}}_{2}\to 2\text{CO} \end{array}$$

(2) $$\begin{array}{r} \text{CO}+3{\text{Fe}}_{2}{\text{O}}_{3}\to {2\text{Fe}}_{3}{\text{O}}_{4}+{\text{CO}}_{2} \end{array}$$

(3) $$\begin{array}{r} {\text{Fe}}_{3}{\text{O}}_{4}+\text{CO }\to 3\text{FeO}+{\text{CO}}_{2} \end{array}$$

(4) $$\begin{array}{r} \text{FeO}+\text{CO }\to \text{Fe}+{\text{CO}}_{2} \end{array}$$

**Figure 2:**
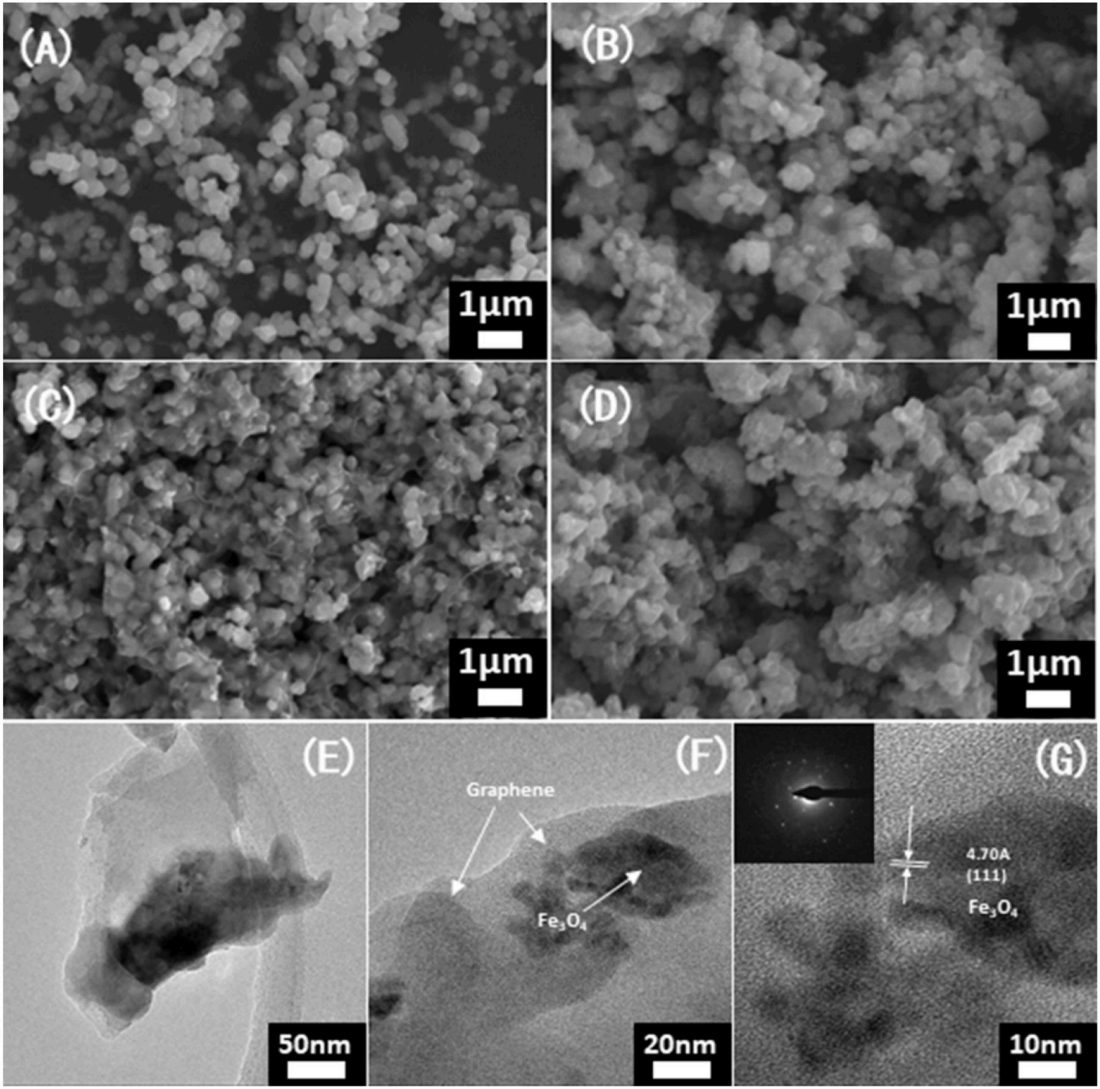
SEM images of **(A)** the initial state for purchased Fe_2_O_3_ particles, **(B)** Fe_3_O_4_@G-500, **(C)** Fe_3_O_4_@G-600, and **(D)** Fe_3_O_4_@G-700. **(E–G)** TEM images and (**G** inset) SAED patterns of Fe_3_O_4_@G-600.

SEM and TEM images further revealed the influence of temperature on morphology. As shown in Figure 2, the Fe_3_O_4_ nanoparticles and graphene layers could be observed. As the carbonization temperature played an important impact on the structure of the Fe_3_O_4_@G, the particles of Fe_3_O_4_@G-500 and Fe_3_O_4_@G-700 aggregated together, while it was obvious that the diaphanous graphene nanosheets coated on particles well in Fe_3_O_4_@G-600 (Figures 2A–F). According to the TGA curves in the Figure S2, the carbon contents of Fe_3_O_4_@G-500, Fe_3_O_4_@G600 and Fe_3_O_4_@G-700 were 23.29, 22.37, and 22.43%, respectively. High-resolution TEM and SAED images confirmed the crystallization of Fe_3_O_4_ nanoparticles (Wang et al., 2013), and lattice fringes with a spacing of 0.47 nm can be seen from the HRTEM image, corresponding to the (111) planes of Fe_3_O_4_ (Figure 2G; Li et al., 2017).

**Figure 3:**
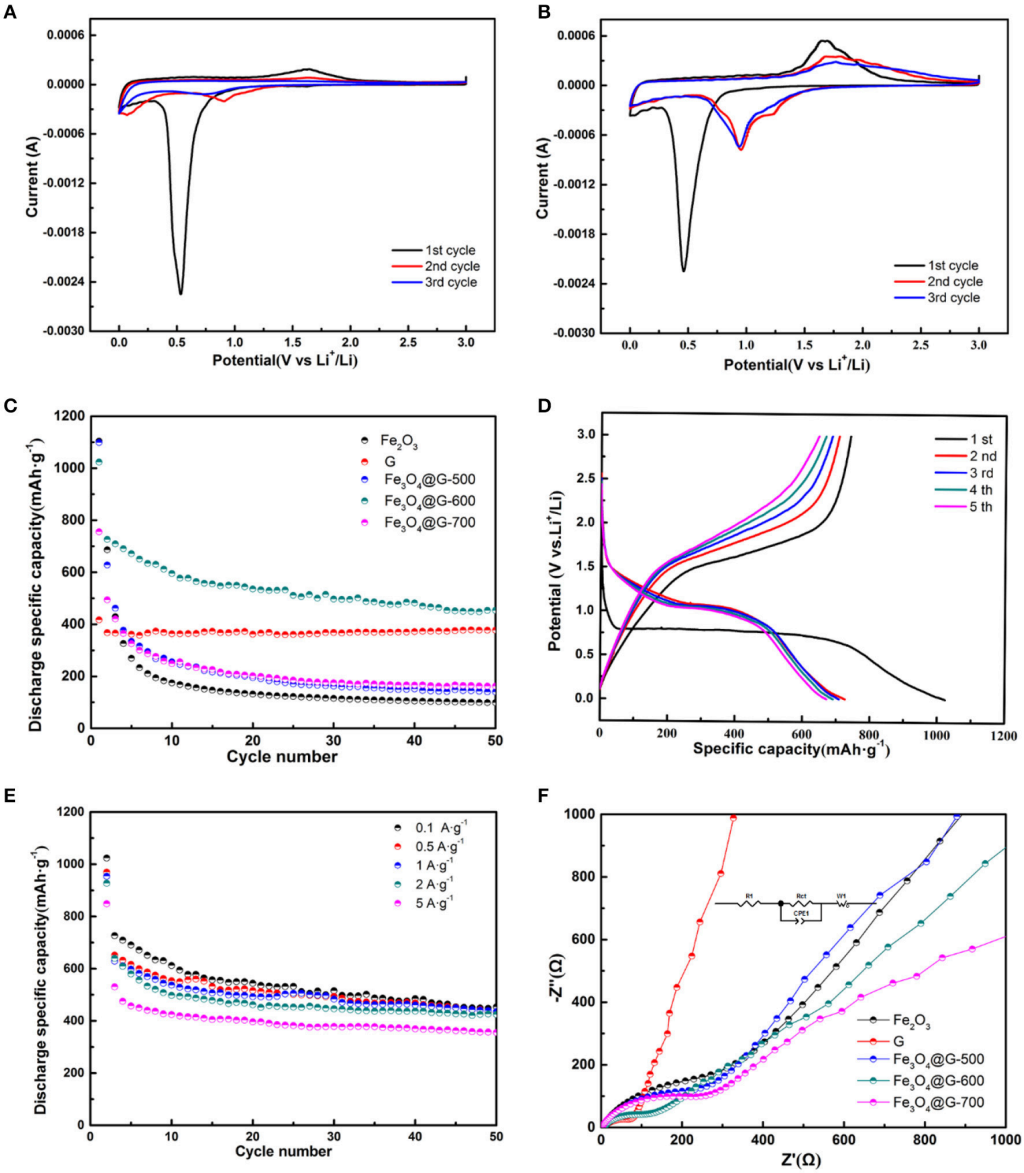
CV curves of **(A)** Fe_2_O_3_ and **(B)** Fe_3_O_4_@G-600 electrodes from the first cycle to the third cycle at a scan rate of 0.1 mV·s^−1^ in the potential range of 0.01 and 3.0 V (vs. Li^+^/Li). **(C)** Cycle performances at 0.1 A·g^−1^ of the Fe_2_O_3_, G, Fe_3_O_4_@G-500, Fe_3_O_4_@G-600, and Fe_3_O_4_@G-700 electrodes. **(D)** The first to fifth galvanostatic charge-discharge profiles and **(E)** rate capabilities of Fe_3_O_4_@G-600 electrode. **(F)** Nyquist plots of the Fe_2_O_3_, G, Fe_3_O_4_@G-500, Fe_3_O_4_@G-600, and Fe_3_O_4_@G-700 electrodes.

CV curves of pure Fe_2_O_3_ and Fe_3_O_4_@G-600 were shown in Figures 3A,B. In Figure 3A, pure Fe_2_O_3_ electrode exhibited a clear cathodic peak at about 0.55 V in the first curve, for the reduction of iron from Fe^3+^ to Fe^0^. In the second and third cycles, the reduction peak at 0.55 V disappeared and the anodic peak at 1.75 V become weak, ascribed to an irreversible phase transformation in the initial cycle (Hassan et al., 2011; Du et al., 2012). As shown in Figure 3B, in the first cycle, the cathodic peak at 0.48 V was attributed to the reversible reduction of Fe_3_O_4_ to Fe^0^ and the irreversible side reactions including the formation of SEI and decomposition of the electrolyte, and the anodic peak at 1.78 V in the reverse anodic scan corresponded to the reversible oxidation of Fe to Fe_3_O_4_ (He C. et al., 2013). However, in the second cycle, the cathodic peak became weak and moved to 0.94 V, which revealed the occurrence of some irreversible reactions and formation of SEI film. It was noted that the third CV curve almost overlapped with the second, exposed a stable reversibility of the composite electrode.

In order to compare the cycle performances of the Fe_2_O_3_, G and Fe_3_O_4_@Gs, the cycle performance at 0.1 A·g^−1^ was investigated as shown in Figure 3C. Compared with pure G (377.3 mAh·g^−1^) and Fe_2_O_3_ (162.7 mAh·g^−1^), the Fe_3_O_4_@G-600 delivered a capacity of 453.6 mAh·g^−1^ after 50 cycles. Fe_3_O_4_@G-600 delivered a high specific capacity of 1,023 mAh·g^−1^ at the first discharge process with a reversible specific capacity of 726.8 mAh·g^−1^, where the initial coulombic efficiency was around 71%. The relatively low initial coulombic efficiency resulted from the irreversible capacity loss for the formation of SEI and decomposition of the electrolyte (Wu et al., 2014).

Figure 3D showed the first to fifth galvanostatic charge-discharge profiles of Fe_3_O_4_@G-600 electrode at 0.1 A·g^−1^. The first discharge voltage curve exhibited one plateau at 0.76 V corresponding to the transformation as described in Equations (5,6) (Wang et al., 2010; Jin et al., 2013). At the same time, charge voltage plateau at about 1.6 V in the initial cycle was attributed to the reversible reactions between Fe_3_O_4_ and Li^+^, The electrochemical reversible reaction mechanism during the charge/discharge processes can be described as Equation (7) (Xia et al., 2013). In comparison, the bare Fe_2_O_3_ electrode displayed poor electrochemical properties because of the poor conductivity and pulverization in lithium intercalation/extraction processes (Figure S3). The electrochemical reversible reaction mechanism of Fe_2_O_3_ during the charge/discharge processes can be given by Equation (8) (Hassan et al., 2011).

(5) $${\text{Fe}}_{3}{\text{O}}_{4}+{\text{xLi}}^{+}+{\text{xe}}^{-}\to {\text{Li}}_{\text{x}}{\text{Fe}}_{3}{\text{O}}_{4}$$

(6) $${\text{Li}}_{\text{x}}{\text{Fe}}_{3}{\text{O}}_{4}+(8-\text{x}){\text{Li}}^{+}+(8-\text{x}){\text{e}}^{-}\to 3{\text{Fe}}^{0}+4{\text{Li}}_{2}\text{O}$$

(7) $${\text{Fe}}_{3}{\text{O}}_{4}+8{\text{Li}}^{+}+8{\text{e}}^{-}↔3{\text{Fe}}^{0}+4{\text{Li}}_{2}\text{O}$$

(8) $${\text{Fe}}_{2}{\text{O}}_{3}+6{\text{Li}}^{+}+6{\text{e}}^{-}↔2{\text{Fe}}^{0}+3{\text{Li}}_{2}\text{O}$$

Rate capabilities of the Fe_3_O_4_@G-600 electrodes were shown in Figure 3E, where the discharge specific capacity is 453.6, 436, 431.2, 424, 355.6 mAh·g^−1^ at 0.1, 0.5, 1, 2, and 5 A·g^−1^ after 50 cycles. In order to confirm the improving electrochemical performance, the EIS was estimated (Figure 3F). The typical Nyquist plots contain a high-frequency semicircle followed by a linear tail in the low-frequency region. And the semicircle corresponded to the charge-transfer resistance (R_ct_). It is obvious that the semicircle for Fe_3_O_4_@G-600 was smallest among Fe_3_O_4_@Gs, suggesting Fe_3_O_4_@G-600 presented the smallest charge transfer resistance (R_ct_) (Wu et al., 2017). EIS measurements were fitted and the parameter results were listed in Table S1. Surface morphology of the Fe_3_O_4_@G-600 electrode also revealed the remarkable cycle stability after 5 and 50 cycles (Figure S4).

According to the results above, Fe_3_O_4_@G-600 presented remarkable performance. Owing to the N-doped graphene coating, it can not only accommodate the volume change but also inhibit the aggregation of Fe_3_O_4_ particles. Meanwhile, the unique two-dimensional graphene nanosheets can promote rapidly electron transport and maintain the structural integrity during the electrochemical lithium insertion/extraction reaction so as to enhance the rate capability of the prepared composite electrode. Comparison of the electrochemical performance of ferric oxide anodes for lithium-ion batteries reported recently was shown in Table S3.

## Template activated method to prepare NGC

According to the result of XRD in Figure S2B, activation occurs only when the temperature was beyond 600°C. Although the FeO phase and Fe phase showed relatively inactive to Li^+^ for Li-ion batteries, recently Hu's group reported (Zhao et al., 2017) that metal template is favor to forming carbon materials with high conductivity, compared with metal oxides template, and higher carbonization temperature is also favorable to forming higher conductivity carbonaceous materials. In this regard, Fe_3_O_4_@G-600, Fe_3_O_4_@G-700, Fe_3_O_4_@G-800, and Fe_3_O_4_@G-900 were used to prepare a series of porous N-doped graphene composites (NGCs) by etching the FeO_x_.

**Figure 4:**
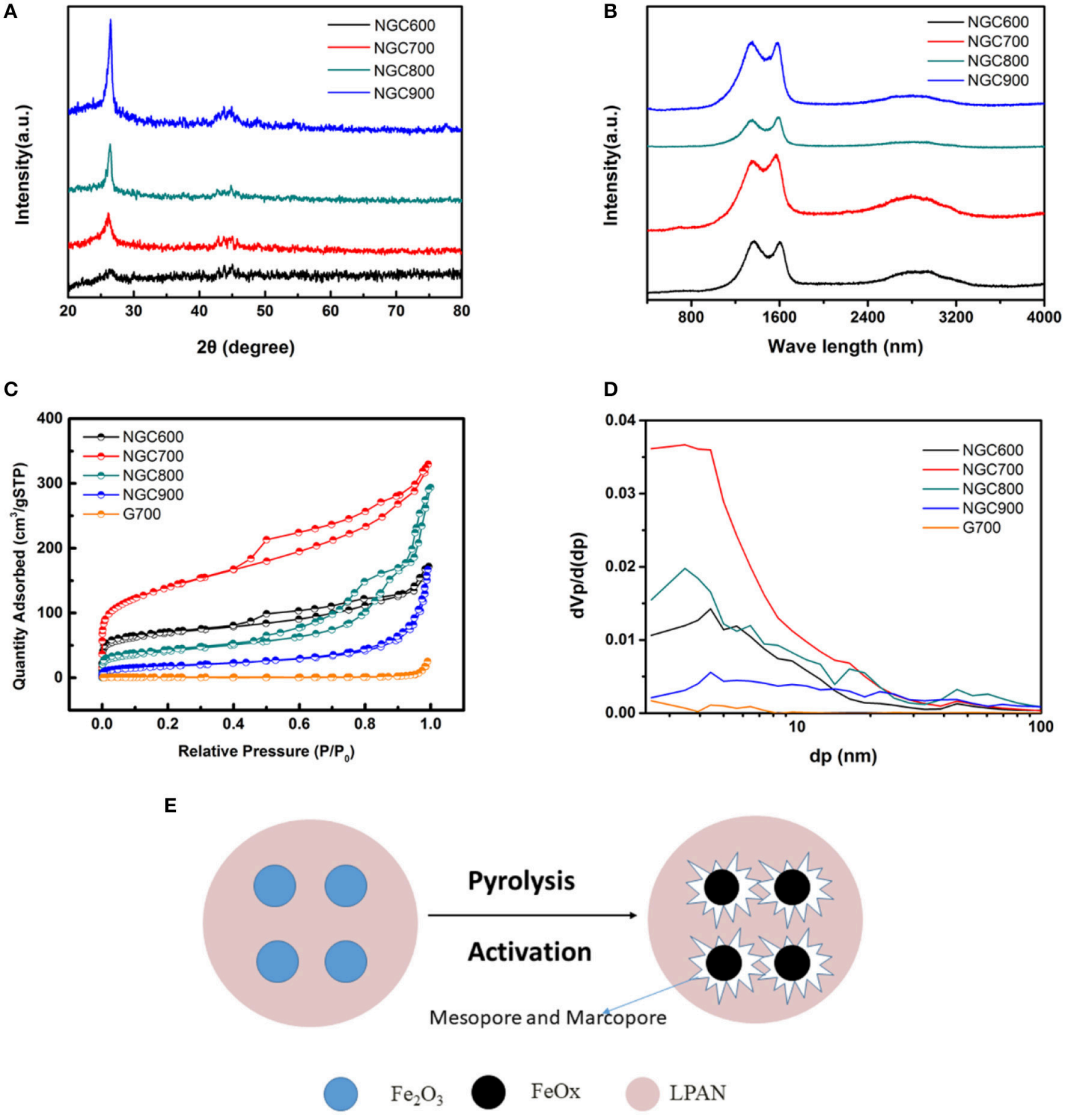
**(A)** XRD patterns and **(B)** Raman spectra of NGCs. **(C)** N_2_ adsorption/desorption isothermal and **(D)** pore size distribution curves of NGC600, NGC700, NGC800, and NGC900. **(E)** Schematic diagram of the activation of porous carbon by FeO_*x*_.

NGCs were estimated to explore the effect of pyrosis temperature on crystallinity and defect degree by X-ray diffraction and Raman spectrum. As shown in Figure 4A, XRD pattern showed two peaks at 2θ = 26.5 and 44.4° of graphene, and the intensity of the peaks increase with a rise of carbonization temperature, which indicated a higher temperature was favorable for the higher crystallinity and graphitization. There was no other peak, indicating that ferric oxide templates were removed completely by HCl. The peaks at 1,360 and 1,590 cm^−1^ corresponded to the D and G bands of graphene, respectively. There was a broad peak of 2D band of carbon at around 2,600–3,100 cm^−1^ (Figure 4B). The broad peak and lower intensity of the 2D band indicated the existence of several graphene layers. The I_D_/I_G_ values which represent the defect quantity were calculated to be 1.036, 0.923, 0.913, and 1.006, respectively. The higher I_D_/I_G_ value could be attributed to the activation of Fe_2_O_3_ to form more defects in the graphene.

N_2_ adsorption-desorption measurements were operated to confirm the specific surface area and pore structure of NGCs. According to the IUPAC classification, the isotherm curves of NGCs belonged to the type IV. And at the P_0_ = 0.4–0.9, a clear hysteresis loop of H4 type was presented, implying the presence of mesopore. However, there was no hysteresis loop on the curve of G700, indicating that there was no mesopore in the G700. The specific surface area was obtained by the Brunauer–Emmett–Teller (BET) equation and the pore size distribution was estimated from the desorption branch of N_2_ isotherms by the Barrett–Joyner–Halenda (BJH) method (Zhao et al., 2015). And the calculation results were summarized in Table 1.

**Table 1:** Textural properties of NGC700.

**NGCs**	**S_BET_ (m^2^·g^−1^)**	**Mesopore (nm)**	**Micropore (nm)**
G700	19.404	2.52	0.69
NGC600	256.87	4.42	0.49
NGC700	505.41	3.47	0.51
NGC800	372.02	10.89	0.69
NGC900	220.69	10.89	0.71

From Figures 4C,D and the calculation results in Table 1, the specific surface area of NGCs were much larger than G700, indicating that the addition of template agent can increase the specific surface area. Obviously, it easy to find that the BET specific surface areas firstly increased from 256.87 m^2^·g^−1^ (NGC600) to 505.41 m^2^·g^−1^ (NGC700), and then decreased to 372.02 m^2^·g^−1^ (NGC800) and 220.69 m^2^·g^−1^ (NGC900). NGC600 presented a small specific area, for Fe_2_O_3_ mainly transform to Fe_3_O_4_ at 600°C (Figure 4C). The reaction between FeO_x_ and carbon materials enhanced with the annealing temperature increasing (from 600 to 700°C), thinning of the channel and resulting in the increase of surface area. Then the specific area of NGC800 and NGC900 decreased because the quantities of tunnels diminished resulting from the further activation of Fe_2_O_3_. Schematic diagram of the activation of porous carbon by FeO_x_ was presented in Figure 4E.

**Figure 5:**
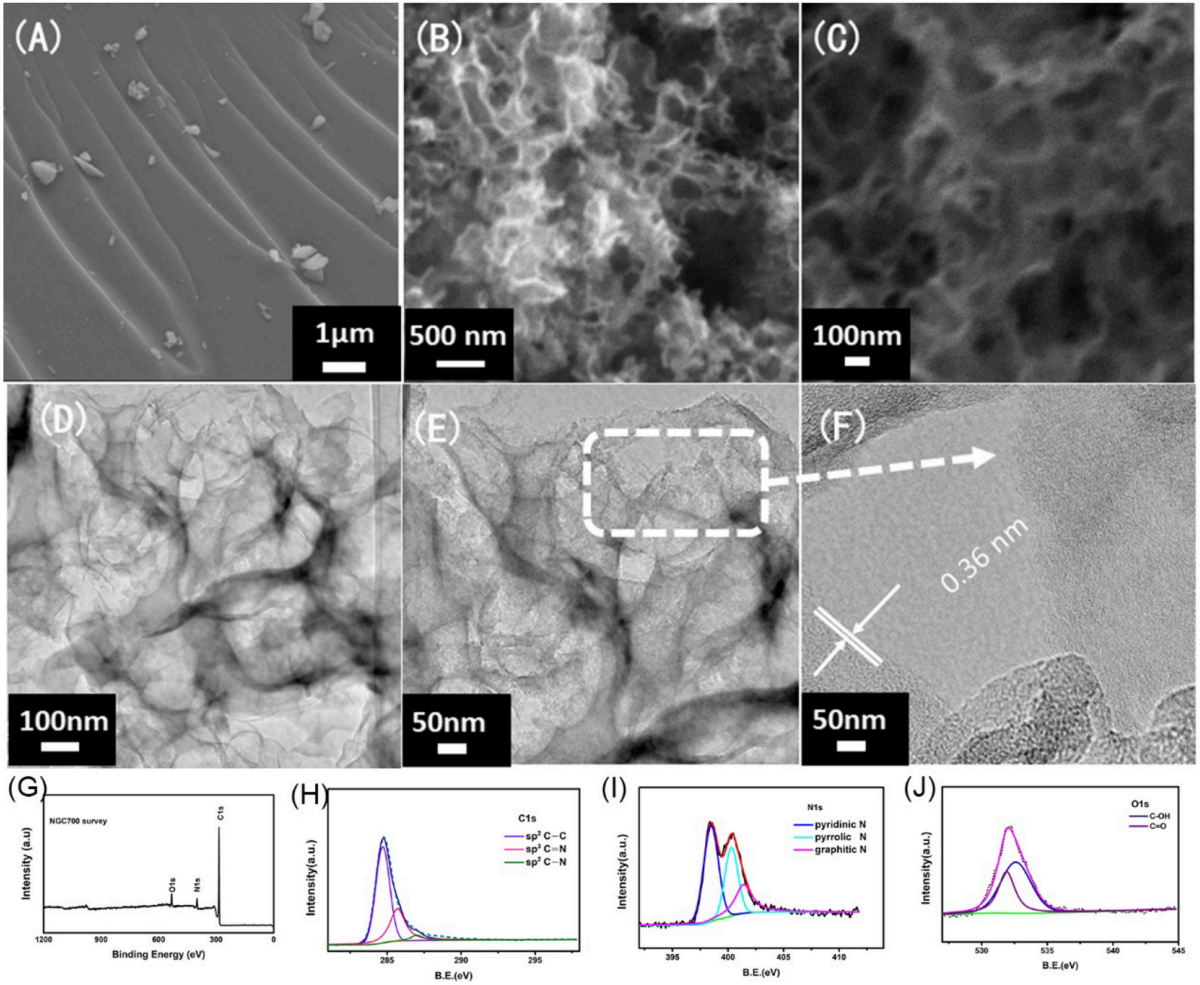
SEM images of the **(A)** G700, **(B,C)** NGC700 at different magnification. **(D–F)** TEM images of the NGC700 at different magnification. XPS spectrum of NGC700: **(G)** Survey spectra, **(H)** C1s XPS, **(I)** N1s XPS, and **(J)** O1s XPS.

The morphology and structure of NGC700 were estimated by SEM and TEM. As shown in Figure 5A, the surface of G700 was smooth without tunnels. On the contrary, NGC700 presented a cellular structure, consisting of abundant pores, which offered a fast path to ion transportation (Figures 5B,C). As shown in Figures 5D–F, the hollow structure could be observed as well as thin graphene layers on the edge. TEM in higher magnification images showed that NGC700 presented graphene lattice with an inter layer spacing of 0.36 nm.

X-ray photoelectron spectroscopy was conducted to further understand the composition of elements and chemical states of the surface (Figures 5G–J). Figure 5G presented three peaks of C (284.5 eV), N (399 eV), and O elements (531 eV) (Mi et al., 2014, 2016; Zhuo et al., 2014). The curves of C, N, and O elements were fitted to understand the existing state of C and N elements in composites. Further analysis of the C1s (Figure 5H) can reveal three peaks: sp^2^C-C (284.6 eV), N-sp^2^C (285.4 eV), and N-sp^3^C (288.4 eV). Most of the carbon atoms form π-π conjugated system, for the main peak at 284.6 eV corresponding to sp^2^ carbon. N 1s (Figure 5I) revealed three peaks: pyridinic N (398.5 eV), pyrrolic N (399.6 eV), and graphitic N (400.5 eV; Zhao et al., 2015). Among these three forms, pyridinic N was viewed as the most suitable for facilitating the electronic conductivity and the charge transfer. The N-doped content was calculated to be 9.52% (Table S2). Ascribed to the O and N-doped in the carbon, the defects increased and the electrochemical activity improved. The polar C-N and C-O on the surface of carbon also increased the wettability of the composites and the contact area of the electrolyte (Tong et al., 2016; Wang et al., 2016). The functional groups on the carbon surface can improve the pseudocapacitance of the materials and the total specific capacitance (Sui et al., 2015; Zhao et al., 2017).

**Figure 6:**
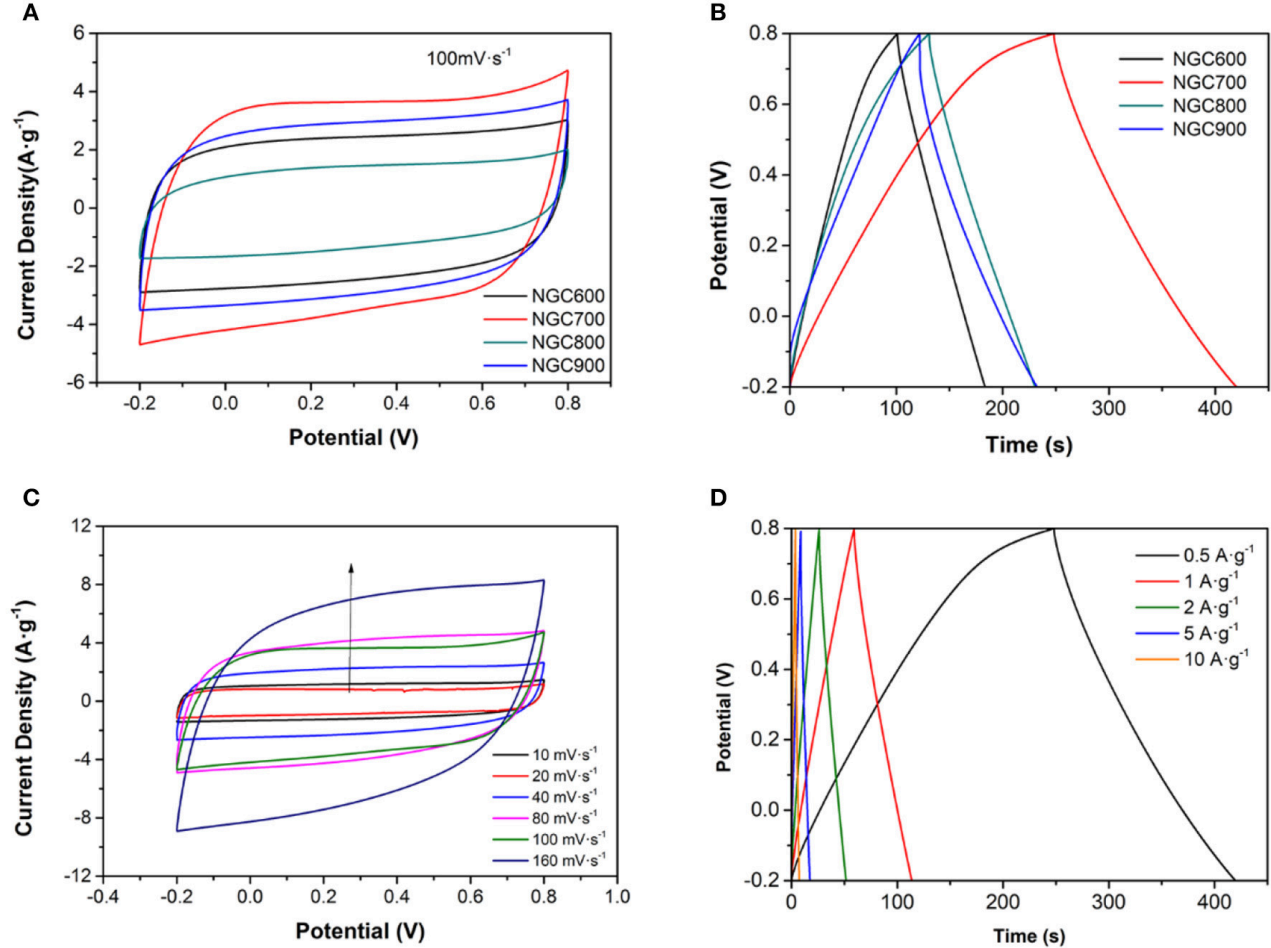
**(A)** CV curves of NGC at the scan rates of 100 mV·s^−1^ **(B)** CP curves of NGC at a current density of 0.5 A·g^−1^. **(C)** CV curves of NGC700 at different scan rates. **(D)** CP curves of NGC700 at various current densities.

To understand the relationship between the structure and the EDLC capacitance performance of the NGCs, cyclic voltammetry (CV), chronopotentiometry (CP) and electrochemical impedance spectroscopy (EIS) were measured in a two-electrode system by coin-type cells. In Figures 6A,C, the CV curve presented a rectangular-like shape at 100 mV·s^−1^ for all samples, which indicated an EDLC capacitive. Chronopotentiometry at the current density of 0.5 A·g^−1^ for samples was in Figure 6B and the specific capacitances of the samples were calculated to be 83, 173, 101, and 122 F·g^−1^, respectively, which were in accordance with the results of the specific surface area above (Table 1). The further electrochemical performance of NGC700 was estimated in Figures 6C,D.

**Figure 7:**
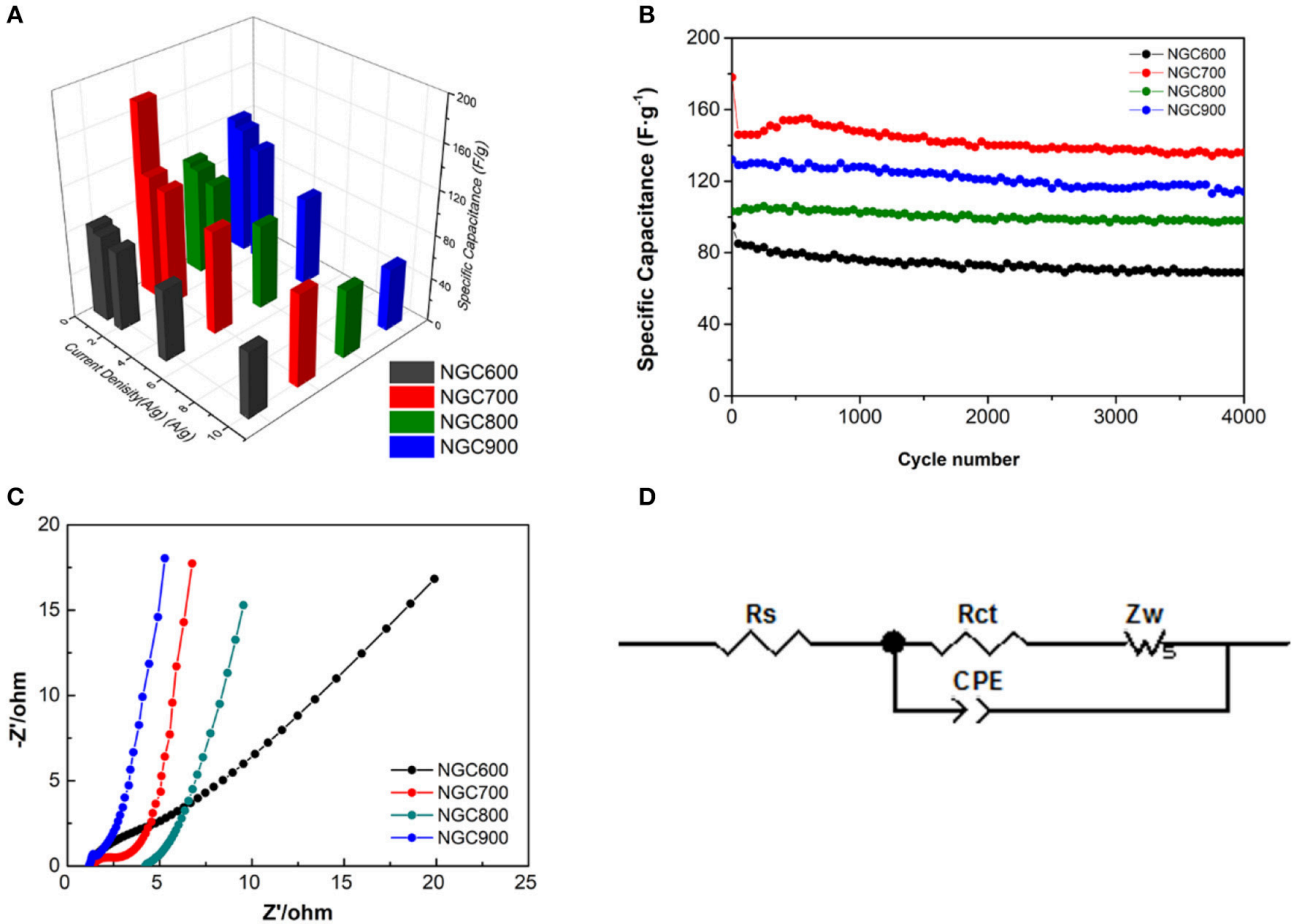
**(A)** The correlation of specific capacitances with scan rates for the composites. **(B)** Cycling performance of NGCs at a current density of 0.5 A·g^−1^. **(C)** Nyquist plots of the NGCs and **(D)** Equivalent circuit of the EIS measurements.

Chronopotentiometry at the current density from 0.5 to 10 A·g^−1^ for other NGCs were also estimated, and the calculation correlation of specific capacitances with scan rates for the composites were shown in Figure 7A. When the current density increases, it was more difficult for ions to diffusion and transfer in the micropore. But the mesopore and macropore were favorable for ion diffusion and transfer at high current density (Zhang and Zhao, 2009). Therefore, with the increase of current density, the specific capacity of NGCs all decreased. Ascribed to suitable mesopore distribution, NGC700 showed well capacitance retaining at high current density, of which the capacitance decreased from 173, 111, 106, 94, and 84 F·g^−1^ at the current density of 0.5, 1, 2, 5, and 10 A·g^−1^, respectively. Generally, the capacitance of materials showed correlation with not only specific surface area but also the pore distribution, conductivity, wettability, etc. The improvement of specific capacitance from NGC600 to NGC700 could be ascribed to its larger specific surface area and conductivity as discussed above. The decrease in capacitance from NGC700 to NGC800 could blame for the same reason. It was worth noting that NGC900 presented higher specific capacitance but lower specific surface area than NGC800. Because high temperature was favor for composites forming high conductivity and more defects. It was consistent with the test results of Raman and BET. After 4,000 cycles, the specific capacitance of NGC700 kept at a stable level and delivered a capacity of 150 F·g^−1^ at the current density of 0.5 A·g^−1^, indicating excellent cycle performance (Figure 7B).

Electrochemical impedance spectroscopy further verified our speculation above. As shown in the Nyquist plot, there was a partial semicircle at high frequency and a vertical straight at the low frequency in each plot. In the high-frequency area, the partial semicircle corresponding to electrode resistances were 2.202, 1.579, 0.730, and 0.350 Ω (Table 2). It was not difficult to find a downward tendency of the electrode resistance as the carbonization temperature increases, which could be ascribed to that the higher temperature was favorable for the higher crystallinity of graphene, so as to improve the conductivity. At the low frequency, NGC700 showed a nearly tilted line, indicating the electrode possessed of the best ionic conductivity and capacitive performance, which was ascribed to its large specific surface area and suitable pore size distribution. Obviously, NGC700 shows much greater performance than others in the electrochemical characterization. This improvement could be ascribed to the proper pore size distribution of NGC700.

**Table 2:** Kinetic parameters of NGC600, NGC 700, NGC 800, and NGC 900 electrodes.

**Sample**	**Rs (Ω)**	**Rct (Ω)**	**Rw (Ω)**
NGC600	1.515	2.202	6.332
NGC700	1.374	1.579	2.628
NGC800	1.185	0.730	2.514
NGC900	1.193	0.350	2.601

## Conclusions

In summary, the prepared Fe_3_O_4_@G from Fe_2_O_3_/LPAN precursors showed optimal cycle stability and rate capability among these synthetic conditions, which delivered a capacity of 355.6 mAh·g^−1^ at the high current density of 5 A·g^−1^ after 50 cycles. What's more, NGCs were prepared by removing the FeO_x_ template form Fe_3_O_4_@G, which presented high specific surface area and abundant pore structure. Ascribed to the activated template FeO_x_, it was reduced with graphene during carbonization, resulting in not only an increase in mesoporous and micropores but an increase in pore size. As a result, NGC700 performed higher specific capacitance and electrochemical stability, delivering a specific capacitance of 172 F·g^−1^ at 0.5 A·g^−1^ current density after 4,000 cycles for supercapacitor. Prepared Fe_3_O_4_@G by one step carbothermal reduction method and NGC by activated template method in one approach, should be very worthy for consideration. Significantly, the raw materials Fe_2_O_3_ is abundant in the earth, so the low cost will guarantee the prospect of the products promising for the next generation LIB and other application in energy storage.

## Author contributions

HM and JL contributed conception and design of the study. HM, XY and JH did the experiments, organized the database and performed the statistical analysis. HM and XY wrote the first draft of manuscript. HM and QZ revised the manuscript. All authors contributed to manuscript revision, read and approved the submitted version.

### Conflict of interest statement

The authors declare that the research was conducted in the absence of any commercial or financial relationships that could be construed as a potential conflict of interest.
