# Supplementary material for: Carbothermal Synthesis of Nitrogen-Doped Graphene Composites for Energy Conversion and Storage Devices
Source: Front Chem. 2018 Oct 22;6:501. doi: 10.3389/fchem.2018.00501 (PMC6204351; doi:10.3389/fchem.2018.00501)
Supplement: Supplementary file 1 [file Table_1.DOCX]

**Supplemental Information**

**Carbothermal Sy****nthesis of N****itrogen-Doped Graphene Composites for Energy Conversion and Storage Devices**


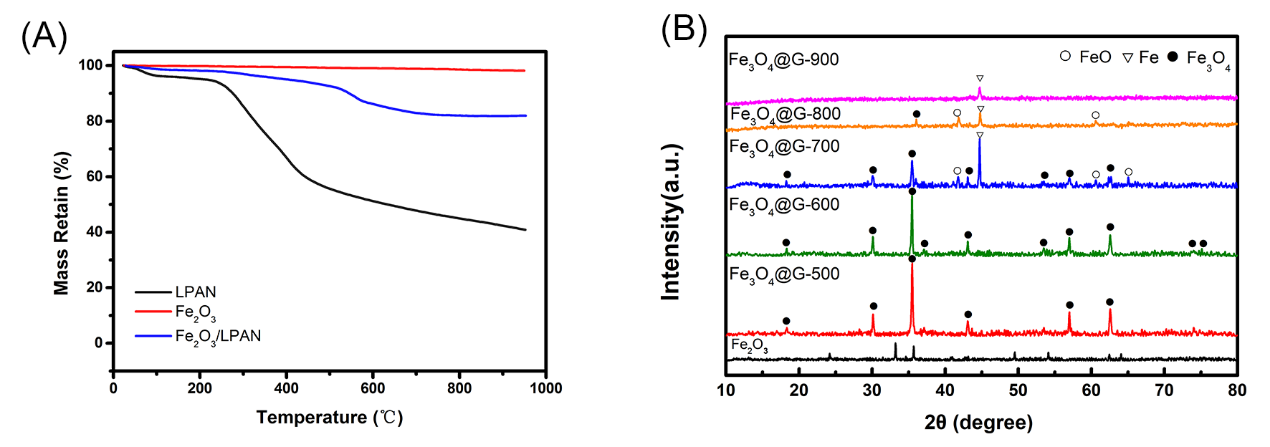


Fig. S1 (A) TG curves of LPAN, bare Fe_2_O_3_ and Fe_2_O_3_-LPAN precursors heated at 220℃ in the air for 3h. (B) XRD patterns of Fe_2_O_3,_ and Fe_3_O_4_@G composites prepared at 500℃, 600℃, 700℃, 800℃ and 900℃ in argon gas.


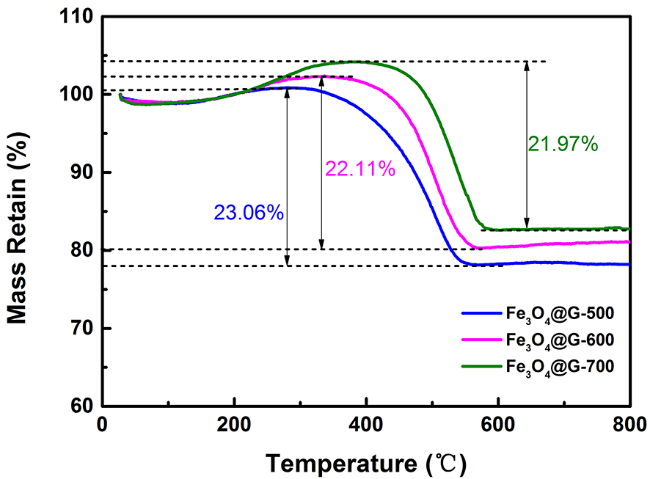


Fig. S2 TG curves of Fe_3_O_4_@G composites in air with a heating rate of 10 ^o^C min^-1^.

The thermogravimetric analysis was also used to calculate the carbon contents in the composites As shown in Fig S2, at first, there was a faint weight loss under 110 °C, owing to evaporation of the water. Secondly, a small weight gain occurred between 110 and 380 °C, for the oxidation of Fe_3_O_4_ to Fe_2_O_3_ in air (Liu et al., 2013; Hu et al., 2014). The final weight loss corresponded to violent oxidation–decomposition of the graphene in the composites. Accordingly, the carbon contents of Fe_3_O_4_@G-500, Fe_3_O_4_@G600 and Fe_3_O_4_@G-700 were 23.29%, 22.37% and 22.43%, respectively.





Fig. S3 The first to fifth galvanostatic charge-discharge profiles of Fe_2_O_3_ electrode between 0.01 and 3.0V at 0.1A·g^-1^.

Table S1 Kinetic parameters of Fe_2_O_3_, G, Fe_3_O_4_@G-500, Fe_3_O_4_@G-600 and Fe_3_O_4_@G-700 electrodes.

| Sample | Rs(Ω) | Rct(Ω) |
| --- | --- | --- |
| Fe_2_O_3_ | **1.134** | **322.6** |
| G | **1.766** | **117.6** |
| Fe_3_O_4_@G-500 | **4.955** | **328.4** |
| Fe_3_O_4_@G-600 | **1.749** | **118.0** |
| Fe_3_O_4_@G-700 | **2.050** | **310.7** |

**
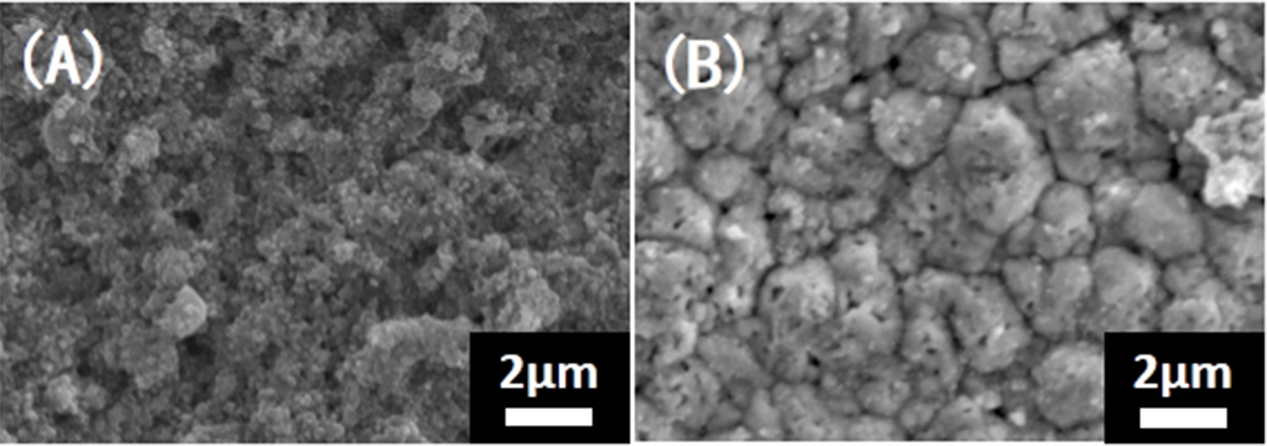
**

Fig. S4 SEM images of the Fe_3_O_4_@G-600 electrode surfaces after (A) 5 and (B) 50 cycles, respectively.

Table S2 Element content of NGC600

| Elements | C | N | O |
| --- | --- | --- | --- |
| Content (%) | 83.31 | 9.52 | 7.17 |

Table S3 Electrochemical performance of iron oxide anodes for lithium-ion batteries.

| Material | Synthesis Method | Fe_3_O_4_ Content  (%) | Cycle Performance | | | Ref. |
| --- | --- | --- | --- | --- | --- | --- |
|  |  |  | Current Density | Cycle Number | Discharge Capacity |  |
| Fe_3_O_4_@Graphene | Ball Milling;  Carbothermal Reduction. | 77.63 | 5 A·g^-1^ | 50 | 355.6 mAh·g^-1^ | This Work |
|  |  |  | 0.1 A·g^-1^ | 50 | 453.6 mAh·g^-1^ |  |
| Fe_3_O_4_@C | Three-step Method | 70 | 0.3 A·g^-1^ | 100 | 254 mAh·g^-1^ | (Liu et al., 2013) |
| Nanorod-like Fe_2_O_3_/graphene | Low-temperature Hydrothermal | 59.6 | 0.1 C | 30 | 404.6 mAh·g^-1^ | (Bing et al., 2014) |
| Fe_3_O_4_/C Nanosheets | [Annealing](https://www.sciencedirect.com/topics/materials-science/annealing) Iron Alkoxide | 89 | 0.5 A·g^-1^ | 100 | 317 mAh·g^-1^ | (Xin et al., 2017) |
| Fe_3_O_4_/C Nanocomposite | Carbothermal Reduction. | N/A | 0.1 A·g^-1^ | 100 | 430 mAh·g^-1^ | (Wang et al., 2013) |
| Fe_3_O_4_/C Nanocomposite | Solution Combustion | 81.4- | 0.1 A·g^-1^ | 100 | 471 mAh·g^-1^ | (Hu et al., 2014) |
| Fe_3_O_4_/rGO | Sodium ascorbate  chelate iron ions | 44.9 | 1 A·g^-1^ | 100 | 300 mAh·g^-1^ | (Liang et al., 2016) |

**Reference**

Bing, Z., Liu, R., Cai, X., Zheng, J., Wu, M., Ling, X., et al. (2014). Nanorod-like Fe_2_O_3_/graphene composite as a high-performance anode material for lithium ion batteries. *J. Appl. Electrochem.* 44(1)**,** 53-60. doi: 10.1007/s10800-013-0599-1.

Hu, M., Jiang, Y., and Yan, M. (2014). Scalable synthesis of Fe_3_O_4_ /C composites with enhanced electrochemical performance as anode materials for lithium-ion batteries. *J. Alloys Compd.* 582(52)**,** 563-568. doi: 10.1016/j.jallcom.2013.08.098.

Liang, C.-L., Liu, Y., Bao, R.-Y., Luo, Y., Yang, W., Xie, B.-H., et al. (2016). Effects of Fe_3_O_4_ loading on the cycling performance of Fe_3_O_4_/rGO composite anode material for lithium ion batteries. *J. Alloys Compd.* 678**,** 80-86. doi: 10.1016/j.jallcom.2016.03.274.

Liu, J., Ni, J., Zhao, Y., Wang, H., and Gao, L. (2013). Grapecluster-like Fe_3_O_4_@C/CNT nanostructures with stable Li-storage capability. *J. Mater. Chem. A* 1(41)**,** 12879-12884. doi: 10.1039/c3ta13141f.

Wang, P., Gao, M., Pan, H., Zhang, J., Liang, C., Wang, J., et al. (2013). A facile synthesis of Fe_3_O_4_/C composite with high cycle stability as anode material for lithium-ion batteries. *J. Power Sources* 239(239)**,** 466-474. doi: 10.1016/j.jpowsour.2013.03.073.

Xin, Q., Gai, L., Wang, Y., Ma, W., Jiang, H., and Tian, Y. (2017). Hierarchically structured Fe_3_O_4_/C nanosheets for effective lithium-ion storage. *J. Alloys Compd.* 691**,** 592-599. doi: 10.1016/j.jallcom.2016.08.331.
